# Relationship between peer evaluation and interprofessional self-evaluation in a joint healthcare team-based learning class involving three universities

**DOI:** 10.20407/fmj.2019-017

**Published:** 2020-07-14

**Authors:** Sayuri Nakamura, Mihoko Itoh, Yoichiro Miki, Toshiaki Kido, Hiroyuki Kamei, Shigetaka Suzuki, Masatsugu Ohtsuki

**Affiliations:** 1 Faculty of Nursing, Fujita Health University, School of Health Sciences, Toyoake, Aichi, Japan; 2 Faculty of Rehabilitation, Fujita Health University, School of Health Sciences, Toyoake, Aichi, Japan; 3 Faculty of Arts and Science, Kyushu University, Fukuoka, Fukuoka, Japan; 4 Faculty of Social Welfare, Department of Social Welfare, Nihon Fukushi University, Chita, Aichi, Japan; 5 Faculty of Pharmacy, Meijo University, Nagoya, Aichi, Japan; 6 Faculty of Medicine, Fujita Health University, School of Medicine, Toyoake, Aichi, Japan

**Keywords:** Interprofessional education, Team-based learning, Peer evaluation, Interprofessional self-evaluation, Medical and health care

## Abstract

**Objective:**

This study aimed to clarify the relationship between interprofessional self-evaluation and peer evaluation during interprofessional education (IPE) using team-based learning (TBL). We also aimed to clarify differences in interprofessional cooperation between students with high and low peer evaluation scores.

**Methods:**

In total, 483 students (grades 3–5) from nine faculties at three universities participated in a TBL-based IPE program. The students completed five interprofessional self-evaluation domains (the modified Tsukuba IPE model) before and after IPE. Students also completed peer evaluation after IPE. Students were divided into three groups by peer evaluation scores (low, middle, high), and the post-class self-evaluation scores of these groups were compared using a Kruskal–Wallis test. Multiple regression analysis was also performed. Peer evaluation comments were analyzed using a qualitative inductive method.

**Results:**

Students in the low peer evaluation group had significantly lower scores in the “Regarding participation in group work” domain than students in the high group (*P*<0.05). Students in the high group received positive comments, such as [good communication] and [working cooperatively], whereas students in the low group were required to improve in two areas: [speaking up more] and [need more communication].

**Conclusions:**

There was a significant relationship between peer evaluation by team members and self-evaluation for “Regarding participation in group work.” Students with high peer evaluation scores participated with active attitudes, whereas students with low scores were considered passive. This study suggested that using peer evaluation may enhance students’ professional cooperation by improving their communication and attitudes toward active participation.

## Introduction

Medical facilities handle various problems, often with complications; therefore, it is no longer sufficient for health workers to be professional, as the current global climate means they also need to be interprofessional.^1^ Many professions are involved in healthcare. However, issues and obstructions arise because of poor interaction and communication between different professional groups.^2,3^ For example, that insufficient information sharing and communication disrupt patient-centered healthcare.^4–6^ It is necessary for multiple professions to cooperate as a team and set common goals for patient-centered healthcare to offer good quality medical care services. Skills involving interactions between different professions often cannot be achieved immediately after a student graduates, meaning it is important to implement phased interprofessional education (IPE) while students are still at university.^1^ Therefore, it is necessary to construct an IPE curriculum for medical and healthcare professional schools.^7^

Fujita Health University is a general health university that has seven undergraduate faculties: medicine, medical technology, nursing, radiological technology, rehabilitation, clinical engineering, and medical management and information science.^8^ Since the university was founded in 1971, it has offered a series of classes called “Assembly,” where students from all faculties participate. Students’ professional cooperation ability is gradually developed through activities in the Assembly classes toward a common goal. In Assembly I, which is held in the first grade, a program is implemented to develop students’ communication skills that form the foundation of professional cooperation. In Assembly II, which is held in the second grade, students work in mixed faculty teams to learn the importance of teamwork. In Assembly III, which is for the third grade and above, teams are formed and presented with complicated cases to consider the most desirable care for the patient.^9^

We noted the potential of the team-based learning (TBL) method^10^ in designing Assembly III classes. TBL is reported to nurture problem-solving skills,^11^ autonomy, willingness, and responsibility.^12^ Therefore, we assumed TBL would be an optimal education method for a class such as Assembly III, and implemented this method into the coursework in 2013. Students from medical-related faculties from other universities have also joined the class since 2016, meaning we conduct large-scale TBL-based IPE for more than 500 students.^13^

We previously reported that the interprofessional self-evaluation scores for professional collaboration improved after the Assembly III class compared with before the class (data were analyzed for the Assembly III class held in 2013).^14^ However, we believed that it was necessary to increase the objectivity of the measurement by using both peer evaluation and subjective self-evaluation. We also assumed that conducting peer evaluation would be useful to understand students’ professional collaboration status and offer feedback for educational purposes. TBL typically involves a preparation process, an individual readiness assurance test, a team readiness assurance test, application exercises, and peer evaluation as an active learning process.^15^ Peer evaluation is an objective evaluation that team members perform for one another, and usually consists of quantitative and qualitative evaluation.^10,16^ The validity and reliability of this approach have been confirmed.^17^ In TBL, it is necessary for each team member to take responsibility and contribute to the team. Therefore, peer evaluation is a key TBL strategy to encourage students to contribute to the group and prevent social indifference (e.g., “I do not need to do it since somebody else can do it”).^16^ This suggests that receiving a high peer evaluation score means that a student fulfilled their duties and contributed to the team. Conversely, a low score reflects a low contribution to the team by that student. In peer evaluation, it is not realistic that everyone is perceived as contributing equally, but peer assessment can be used to improve non-uniform distribution.^12^

Several previous studies reported a correlation between peer evaluation scores and academic record indicators.^18–21^ Therefore, we thought that peer evaluation may also be correlated with interprofessional self-evaluation. Peer evaluation has been reported to have five purposes: assessment and learning tool, installation of social control in the learning environment, preparation of students for self-monitoring and self-regulation in lifelong learning, and active participation.^22^ If there is a relationship between peer evaluation and interprofessional self-evaluation, we believe that using related factors could provide suggestions for effective professional education.

The objective of this study was to clarify the relationship between interprofessional self-evaluation (a subjective evaluation) and peer evaluation by others (an objective evaluation). Furthermore, we divided participating students into three groups based on peer evaluation scores, and clarified differences in the characteristics of professional cooperation between students with high and low peer evaluation scores. By clarifying these characteristics, we hope to develop suggestions for educational approaches that will encourage students to cooperate with each other and contribute to healthcare.

## Methods

### 1. Participants

Students in grades 3–5 from nine faculties at three universities were chosen as research participants: medicine, medical technology, nursing, radiological technology, rehabilitation, clinical engineering, medical management and information science, pharmacy, and social welfare. Of the 583 registered students, 581 who attended all three Assembly III classes were included as participants in this study; two students that did not attend any of the Assembly III classes were excluded.

### 2. Interprofessional education

In this study, IPE comprised three days of Assembly III classes: 4 hours on June 7, 2017; 4 hours on June 16, 2017; and 3 hours on June 23, 2017. The teaching method used was TBL. Each group comprised 5–6 students from different faculties, with a total of 94 groups. The groups were spread across four classrooms: two classrooms each held 28 groups, one classroom held 24 groups, and one classroom held 18 groups. Each classroom had seven teachers who managed the class. The class topic was “How a patient with dementia and their family can continue to live in their home and community where they have lived for a long time without any problems.” The course material was developed and reviewed by 37 teachers from across all faculties. About 1 week before the class, students attended an orientation session where they completed a pre-class interprofessional self-evaluation. Preparatory materials were also handed out during this session.

On the first day of class, students completed an individual readiness assurance test followed by a team readiness assurance test to determine how well they had prepared. In this class, students could also appeal any questions they had gotten wrong but that they thought were correct. In addition, they received feedback from teachers, and performed application activities. The application problems for the first day were “identify potential problems that a patient with dementia and their family would face” and “discuss what can be done to solve such problems.” After discussion within the teams, discussions were held between the teams. On the second day of class, students practiced application activities, presented their opinions and voted for the best presentation, performed peer evaluation, and completed post-class interprofessional self-evaluation pertaining to interprofessional cooperation. Peer evaluation scores and anonymized comments from team members were later returned to the students. As the objective of these peer evaluations was formative assessment, the scores did not reflect students’ grades for the class. The application activity for the second day was “discuss how a patient with dementia and their family can continue to live in their home and community where they have lived for a long time without any problems.” Teams were required to present their findings in front of the class using a poster. On the third day of class, all students gathered in an auditorium and listened to the 11 the best presentations as voted in the previous class.

### 3. Measures

#### 1) Interprofessional self-evaluation

The interprofessional self-evaluation scale used in this study included the most recent Interprofessional Education Tsukuba (IPET) model (38 items).^23^ The correlation coefficient between the overall IPET scale and the Readiness for Interprofessional Learning Scale was 0.55, indicating the IPET had criterion-related validity. In addition, Cronbach’s alpha coefficients for each category in the IPET scale were 0.84–0.92, indicating reliability.^23^ As the original scale did not include some of the professions that are developed in our university, we added two items pertaining to these professions (clinical engineer and medical information management officer), giving a total of 40 items. These two items were “I think I understand the role of a clinical engineer” and “I think I understand the role of a medical information management officer.” These 40 interprofessional self-evaluation items were categorized into five domains: 1) feelings about the profession I am training for (eight items); 2) understanding the role of each profession’s specialization (12 items); 3) regarding participation in group work (six items); 4) thoughts regarding the team in healthcare and welfare (10 items); and 5) feelings about cooperation among different professions (four items). Each item was measured using a six-point Likert scale: 1 (strongly disagree), 2 (disagree), 3 (somewhat disagree), 4 (somewhat agree), 5 (agree), and 6 (strongly agree).

The 40 items and five domains were translated into English by a native English speaker who was familiar with the Japanese language. The developer of the scale then reviewed the translated version and made revisions in collaboration with the translator to ensure the English version was as close to the original version as possible.

#### 2) Peer evaluation

Quantitative and qualitative evaluations were performed according to Michaelson’s method.^10,16^ We used a quantitative evaluation method in which students were evaluated by other members of their team and allocated points based on their contribution. Students were instructed to determine differences in contributions so that all team members would not have the same number of points. The total contribution was set at 100 points, and each team member was given a number of points depending on how much they contributed. Therefore, individual scores were calculated by dividing by 100 points among the team members, meaning the average contribution score was 1.0. Comments written by each team member were used for the qualitative evaluation. According to methods used in previous studies,^10,16^ we used two qualitative evaluation items: positive comments and critical comments. We explained to the students that their comments should show respect for their team members rather than any blame. These comments were input using a computer to prevent students from being identified by their handwriting. Peer evaluations were compiled for each individual and then returned to students to encourage their awareness and growth. Because the comments were randomized on the computer, it was impossible to determine from which team member a comment was received.

### 4. Statistical analysis

The correlation between the post-class interprofessional self-evaluation total score and the peer evaluation score was determined by using Spearman’s rank correlation analysis. Furthermore, the peer evaluation scores were categorized into three groups: a low group (less than 0.83), a middle group (between 0.83 and 1.06), and a high group (more than 1.06). The post-class interprofessional self-evaluation overall, domain, and item scores for the three groups were compared using Kruskal–Wallis tests, because they were not normally distributed. Thereafter, multiple comparisons were performed using the Dunn test. In addition, a multiple regression analysis was performed to confirm factors that affected peer evaluation. Peer evaluation was the dependent variable, and attributes (sex, age, faculty) and interprofessional self-evaluation domains were the independent variables.

The analyses were performed using SPSS version 25.0 (IBM Corp., Armonk, NY), with the significance level set at 0.05. Comments written in the peer evaluation were analyzed using a qualitative inductive method.^24^ The comments for the low and high peer evaluation groups were divided into positive and critical comments. Next, these comments were analyzed by extracting one-definition units and coded, and then further categorized based on similarities. The coding and categorization were repeated among researchers to ensure validity.

### 5. Ethical Considerations

The objective, methods, and content of the interprofessional self-evaluation scale were verbally explained to participating students. The students were also told that their interprofessional self-evaluation data would be used for this study, and that they could choose whether to participate in the study. They were informed that there would be no consequences for refusing to participate. Return of a completed scale was deemed provision of consent to participate in this study. The objective, content, and management of the peer evaluation information were explained online via the Assembly education web site. Contact information was included so that any student who decided to withdraw from the study could contact the research team representative via email. All personal information that could be used to determine a student’s identity (e.g., student number and name) was deleted, and each completed response was assigned a random number.

This study was approved by the Fujita Health University Medical Research Ethics Review Committee (Approval number: HM18-250).

## Results

In total, 560 of the 581 students agreed to participate in this study (response rate 96.4%). After excluding 77 students whose responses were incomplete, 483 participants were included in the analysis (valid response rate 86.3%). Participants’ attributes are shown in Table 1. There were 195 male (40.4%) and 288 female students (59.6%). The largest number of students were from the faculty of medicine (n=93, 19.3%), followed by nursing (n=91, 18.8%), medical technology (n=80, 16.6%), clinical engineering (n=55, 11.4%), radiological technology (n=46, 9.5%), rehabilitation (n=44, 9.1%), medical management and information science (n=32, 6.6%), social welfare (n=23, 4.8%), and pharmacy (n=19, 3.9%). The average age of participating students was 21.0±2.2 years.

**Figure 1:**
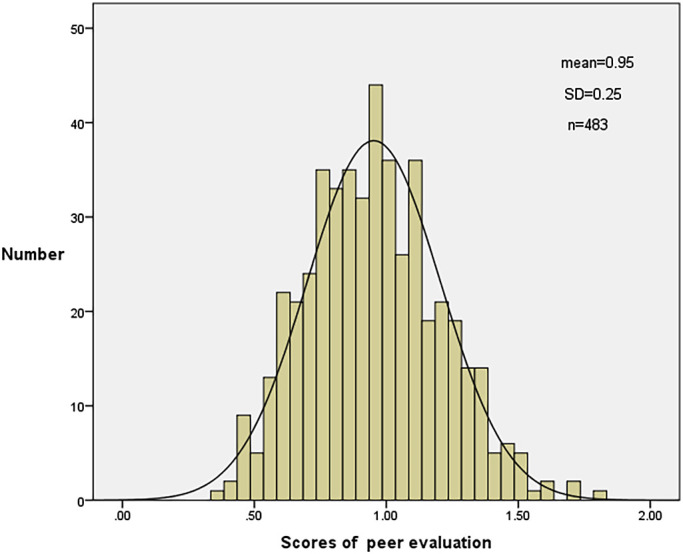
Distribution of peer evaluation scores

In the present study, interprofessional self-evaluation was performed using a 40-item scale, including the 38 original items from the IPET scale and an additional two items. With the two additional items, the Spearman’s coefficients in the correlation analysis between pre-IPE and post-IPE were 0.265–0.653, indicating a significant correlation (p<0.001) and reproducibility. In addition, the Cronbach’s alpha coefficients for the 40-item scale were 0.92–0.97, indicating that reliability was maintained. Furthermore, there was a significant correlation between the scores for the original 38-item IPET scale and our 40-item scale (p<0.001), which indicated our scale had criterion-related validity. The distribution of peer evaluation scores is shown in Figure 1. The mean peer evaluation score was 0.95±0.25.

Participants’ attributes by the three peer evaluation score groups (low, middle, high) are shown in Table 2. We found a significant association between sex and peer evaluation group using a chi-square test (p<0.001), which showed there were more males in the low group, and more females in the middle group. There was also a significant association between faculty and group (p<0.001). Students in the medical technology, clinical engineering, and radiological technology faculties tended to be in the low group, students from the nursing and social welfare faculties tended to be in the middle group, and students from the medicine, rehabilitation, medical management and information science, and pharmacy faculties tended to be in the high group. One-way analysis of variance showed there were no significant differences in age among three peer evaluation groups.

The correlations between the interprofessional self-evaluation scores and the peer evaluation scores are shown in Table 3. Significant correlations were found between peer evaluation scores and the categorized interprofessional self-evaluation scores for domains 3 (r=0.211, p<0.001) and 4 (r=0.103, p=0.024), and the overall score (r=0.120, p=0.009).

The mean total scores for each domain and the post-class interprofessional self-evaluation overall scores were divided into three groups based on the peer evaluation scores (Table 4). There was a significant difference between the three groups in domain 3 “Regarding participation in group work” and overall scores (p<0.001 and p=0.034, respectively); interprofessional self-evaluation scores became higher as peer evaluation scores became higher. There were significant differences in domain 3 of the interprofessional self-evaluation scores between the low and middle peer evaluation groups, and between the low and high groups by multiple comparison (p=0.002 and p<0.001, respectively). There was a significant difference in the overall interprofessional self-evaluation score between the low and high groups (p=0.033).

Each item in domain 3 (“Regarding participation in group work”) showed significant differences (Table 5). There were significant differences between the three groups for: “I speak to convey my thoughts to the other members,” “I actively participate in group work as a member of the group,” “I strive to advance the group work by cooperating with other members,” and “I strive to demonstrate the specialization of my particular profession” (p<0.01). In addition, the interprofessional self-evaluation score became higher as the peer evaluation score became higher. In the first three items, significant differences were observed between the low and middle groups, and between the low and high groups by multiple comparison (p<0.05). The last item showed a significant difference between the low and high groups (p=0.009).

Multiple regression analysis was performed using attributes and each domain of interprofessional self-evaluation as independent variables to confirm the factors affecting peer evaluation (Table 6). Significant associations were found between age and the “Regarding participation in group work” domain (β=0.098, p=0.028; β=0.343, p<0.001, respectively). The adjusted R^2^ was 0.068.

Next, the comments written by the group members were analyzed (Table 7). Sixteen categories emerged in the positive comments from the low peer evaluation group, and 29 categories emerged in the positive comments for the high group (categories denoted using square brackets [ ]). The category that was only present in the low group was [perform tasks when asked]. Categories that were only present in the high group were [rich knowledge], [intelligible explanation], [accurate opinions], [persuasive opinions], [thoughts from the patient’s perspective], [good communication], [offering solutions], [respect of peers’ opinions], [treating all team members equally], [working responsibly], [working cooperatively], [working seriously], [leadership], and [organizing the group].

Similarly, categories of critical comments for the low and high peer evaluation groups are shown in Table 8. Critical comments for the low group were classified into 10 categories, and critical comments for the high group were classified into eight categories. Categories that were only present in the low group were [speak up more] and [need more communication]. No categories were only found in the high group.

## Discussion

In the present study, we found weak but positive correlations between overall interprofessional self-evaluation scores and peer evaluation scores. In addition, the three-group comparison revealed a relationship between overall scores interprofessional self-evaluation scores and peer evaluation scores in the high peer evaluation group. Previous studies reported that peer evaluation was correlated with student evaluations by teachers^25,26^ and the record of doctors’ national examinations.^19,20^ This study revealed that peer evaluation was also related to subjective interprofessional self-evaluation. Multiple regression analysis showed that peer evaluation was affected by domain 3 in our interprofessional self-evaluation scale, which was related to participation attitude in group work. Age also influenced peer evaluation, which suggested that older students might have demonstrated more contribution to the group than younger students.

Communication, interpersonal skills, and teamwork are key health professional competencies.^16^ This study showed that communication skills as represented in a self-evaluation item (“I speak to convey my thoughts to the other members”) were related to peer evaluation. Other items (“I actively participate in group work as a member of the group,” “I strive to advance the group work by cooperating with other members,” and “I strive to demonstrate the specialization of my particular profession”) required interpersonal skills and teamwork. We found that students in the low peer evaluation group were rated low for these competencies.

According to positive comments from team members, students in the high peer evaluation group performed tasks [seriously] and [cooperatively] with fellow members. Students in the high group were perceived to have [rich knowledge] and share ideas with [respect of peer’s opinions], [intelligible explanation], [accurate opinions], and [persuasive opinions], which might contribute to accelerating team development through multiple discussions among team members leading to integrated ideas within the team. In addition, students in the high group showed [good communication], worked [responsibly, and equally], offered [patient’s perspective and solutions], and [organized] the team with [leadership]. However, students in the low group tended to only “perform tasks when asked.” This can be interpreted as meaning that tasks were performed if requested, but not proactively, suggesting that these students did not show an active participation attitude in group work. Previous studies reported there was “active participation” in comments to students with high peer evaluation scores, and “passive participation” in comments to students with low peer evaluation scores.^17^ This finding suggested that active participation in IPE is a priority issue that should be encouraged.

Critical comments from students in the low peer evaluation group included [speaking up more] and [need more communication]. These results indicated that improvement of communication skills was required to lead active discussions among all team members.

A limitation of the present study was that evaluation of interprofessional cooperation was based on self-evaluation, which raises a concern regarding the objectiveness of the results. There were students who selected the same numbers (rating) for all answers; they might have selected the same rating for each item without seriously reading the item. However, there was a relationship between interprofessional self-evaluation and peer evaluation scores, which suggested that interprofessional self-evaluation can be a useful evaluation indicator. We found a significant association between faculty and peer evaluation group when discussing “How a patient with dementia and their family can continue to live in their home and community where they have lived for a long time without any problems.” However, this might have been because students from the medical technology, clinical engineering, and radiological technology faculties (in which the professionals provide patient care indirectly) might have been less able to express their opinions than the other students (e.g., those who provide direct care). However, this study could not elucidate the reason for this association and further research is needed to elucidate this point.

In conclusion, there was a significant relationship between peer evaluation by team members and self-evaluation in terms of participation in group work. Students in the low peer evaluation group had significantly lower scores for “Regarding participation in group work” than students in the high peer evaluation group. Students in the high group received positive reviews from their team members for [rich knowledge], [intelligible explanation], [accurate opinions], [persuasive opinions], [thoughts from the patient’s perspective], [good communication], [offering solutions], [respect of peers’ opinions], [treating all team members equally], [working responsibly], [working cooperatively], [working seriously], [leadership], and [organizing the group]. Conversely, students in the low group were asked by team members to improve [speaking up more] and [need more communication].

This study suggests that using a comparatively simple peer evaluation method may enhance students’ professional cooperation by improving communication and active participation attitudes for students with low peer assessments.

## Figures and Tables

**Table1:** Attributes of study participants (n=483)

		n (%)
Sex	Male	195 (40.4)
	Female	288 (59.6)
Faculty and Grade	Medicine, grade 3	93 (19.3)
	Nursing, grade 3	91 (18.8)
	Medical Technology, grade 4	80 (16.6)
	Clinical Engineering, grade 3	55 (11.4)
	Radiological Technology, grade 3	46 ( 9.5)
	Rehabilitation, grade 3	44 ( 9.1)
	Medical Management and Information Science, grade 4	32 ( 6.6)
	Social Welfare, grades 3–4	23 ( 4.8)
	Pharmacy, grade 5	19 ( 3.9)
		Mean±SD
Age, years		21.0±2.2

SD, standard deviation.

**Table2:** Attributes of participants by peer evaluation score group (n=483)

		Peer evaluation			p-value
		Low n (%)	Middle n (%)	High n (%)	
Sex	Male	79 (40.5)	49 (25.1)	67 (34.4)	<0.001
	Female	80 (27.8)	114 (39.6)	94 (32.6)	
Faculty	Medicine	28 (30.1)	24 (25.8)	41 (44.1)	<0.001
	Nursing	27 (29.7)	33 (36.3)	31 (34.0)	
	Medical Technology	34 (42.5)	28 (35.0)	18 (22.5)	
	Clinical Engineering	20 (36.4)	19 (34.5)	16 (29.1)	
	Radiological Technology	23 (50.0)	19 (41.3)	4 ( 8.7)	
	Rehabilitation	13 (29.5)	13 (29.5)	18 (41.0)	
	Medical Management and Information Science	10 (31.3)	9 (28.1)	13 (40.6)	
	Social Welfare	4 (17.4)	12 (52.2)	7 (30.4)	
	Pharmacy	0 ( 0.0)	6 (31.6)	13 (68.4)	
		Mean±SD	Mean±SD	Mean±SD	
Age, years		20.9±1.2	20.9±2.6	21.4±2.3	0.065

Chi-square test: sex and faculty. One-way analysis of variance: age. SD, standard deviation.

**Table3:** Correlation coefficients between post-class interprofessional self-evaluation and peer evaluation scores (n=483)

Interprofessional self-evaluation	Peer evaluation	
	r	p-value
Domain 1: Feelings about the profession I am training for (8 items)	0.075	0.102
Domain 2: Understanding the role of each profession’s specialization (12 items)	0.002	0.969
Domain 3: Regarding participation in group work (6 items)	0.211	<0.001
Domain 4: Thoughts regarding the team in healthcare and welfare (10 items)	0.103	0.024
Domain 5: Feelings about cooperation among different professions (4 items)	0.088	0.054
Overall (40 items)	0.120	0.009

Spearman’s rank correlation coefficient.

**Table4:** Mean domain and total scores for the post-class interprofessional self-evaluation by peer evaluation score groups

Interprofessional self-evaluation	Peer evaluation			p-value	Multiple comparison
	Low (L) (n=159)	Middle (M) (n=163)	High (H) (n=161)		
	Mean±SD	Mean±SD	Mean±SD		
Domain 1: Feelings about the profession I am training for (8 items)	34.08±7.23	34.98±6.54	35.53±6.67	0.211	
Domain 2: Understanding the role of each profession’s specialization (12 items)	46.11±9.99	45.77±9.83	46.04±9.40	0.848	
Domain 3: Regarding participation in group work (6 items)	25.55±5.45	27.47±4.37	27.99±3.65	<0.001	L<M p=0.002 L<H p<0.001
Domain 4: Thoughts regarding the team in healthcare and welfare (10 items)	47.33±8.02	49.09±6.84	49.32±6.50	0.086	
Domain 5: Feelings about cooperation among different professions (4 items)	18.60±3.33	19.32±2.84	19.34±2.66	0.068	
Total (40 items)	171.67±26.25	176.60±23.70	178.23±21.01	0.034	L<H p=0.033

Kruskal-Wallis test, Dunn test. SD, standard deviation.

**Table5:** Mean post-class interprofessional self-evaluation scores for domain 3 items by peer evaluation score groups

Domain 3: Regarding participation in group work	Peer evaluation			p-value	Multiple comparison
	Low (L) (n=159)	Middle (M) (n=163)	High (H) (n=161)		
	Mean±SD	Mean±SD	Mean±SD		
1. I speak to convey my thoughts to the other members.	4.06±1.03	4.44±0.85	4.58±0.75	<0.001	L<M p=0.004 L<H p<0.001
2. I strive to listen to the opinions of other members.	4.57±0.94	4.80±0.77	4.79±0.72	0.067	
3. I take a flexible attitude when presented with opinions differing from my own.	4.49±0.97	4.67±0.82	4.65±0.72	0.332	
4. I actively participate in group work as a member of the group.	4.06±1.15	4.60±0.83	4.77±0.82	<0.001	L<M p<0.001 L<H p<0.001
5. I strive to advance the group work by cooperating with other members.	4.28±1.07	4.60±0.82	4.76±0.75	<0.001	L<M p=0.045 L<H p<0.001
6. I strive to demonstrate the specialization of my particular profession.	4.09±1.08	4.36±0.97	4.43±0.89	0.009	L<H p=0.009

Kruskal-Wallis test, Dunn test. SD, standard deviation.

**Table6:** Multiple regression analysis of peer evaluation scores (n=483)

Variable	β	p-value
Sex	0.060	0.185
Age	0.098	0.028
Faculty	0.022	0.628
Domain 1: Feelings about the profession I am training for	–0.060	0.291
Domain 2: Understanding the role of each profession’s specialization	–0.093	0.060
Domain 3: Regarding participation in group work	0.343	<0.001
Domain 4: Thoughts regarding the team in healthcare and welfare	0.002	0.983
Domain 5: Feelings about cooperation among different professions	–0.066	0.399
R^2^	0.084	
Adjusted R^2^	0.068	

**Table7:** Categories of the positive comments in the low and high peer evaluation groups

Categories in the low group	Categories in the high group
Preparation	Preparation
Searching information during group work	Searching information during group work
—	Rich knowledge
Own thoughts	Own thoughts
Have opinions	Have opinions
Professional opinion	Professional opinion
—	Intelligible explanation
—	Accurate opinions
—	Persuasive opinions
—	Thoughts from the patient’s perspective
Novel opinions	Novel opinions
—	Good communication
—	Offering solutions
Summary of the thoughts of the group	Summary of the thoughts of the group
Listening to others’ opinions	Listening to others’ opinions
—	Respect for peers’ opinions
How one listens to others talking	How one listens to others talking
—	Treating all team members equally
Creating a friendly atmosphere	Creating a friendly atmosphere
Perform tasks to be asked	—
—	Working responsibly
—	Working cooperatively
Volunteering for the role of secretary	Volunteering for the role of secretary
Volunteering for the role of the presenter	Volunteering for the role of the presenter
Volunteering for the role of facilitator	Volunteering for the role of facilitator
—	Working seriously
Active participation	Active participation
Performing multiple roles	Performing multiple roles
—	Leadership
—	Organizing the group

**Table8:** Categories of critical comments in the low and high peer evaluation groups

Categories in the low group	Categories in the high group
Need to prepare more	Need to prepare more
Need to say one’s opinion more	Need to say one’s opinion more
Need to think more before talking	Need to think more before talking
Speak up more	—
Be more confident	Be more confident
Difficult to understand	Difficult to understand
Need more professional opinions	Need more professional opinions
Need to listen more	Need to listen more
Need to actively participate	Need to actively participate
Need more communication	—
